# Supplementary material for: Phase-field modeling of colloid-polymer mixtures in microgravity
Source: NPJ Microgravity. 2025 Sep 1;11:62. doi: 10.1038/s41526-025-00500-0 (PMC12402263; doi:10.1038/s41526-025-00500-0)
Supplement: Supplementary file 1 — Supplementary Information [file 41526_2025_500_MOESM1_ESM.pdf]

Supplementary Information:
Phase-field modeling of colloid-polymer mixtures in microgravity

Lauren Barnes[1], Boris Khusid[2], Lou Kondic[1], William V. Meyer[3] and Anand U. Oza[1,*]

[1] Department of Mathematical Sciences & Center for Applied Mathematics and Statistics, New Jersey Institute of Technology, Newark, New Jersey 07102, USA

[2] Otto H. York Department of Chemical and Materials Engineering, New Jersey Institute of Technology, Newark, New Jersey 07102, USA

[3] USRA at NASA Glenn Research Center, Cleveland, Ohio 44135, USA

[*]corresponding author

DETAILS OF BCAT-3 AND BCAT-4 EXPERIMENTS

We here collect the processed images for the BCAT-3 and BCAT-4 experiments. The parameter values are given in Table I of the Main Text. Supplementary Figures 1–4 show the time evolution of the four BCAT-3 samples, and Supplementary Figs. 5–6 shows that for two of the BCAT-4 samples. We did not include a figure for BCAT-4 Sample 1, for which only a few images were available, likely because the camera malfunctioned during the experiment.

Examples of the azimuthally-averaged autocorrelation function $\tilde{C}(r)$, as defined in the "Results" section of the Main Text, are shown in Supplementary Fig. 7 for BCAT-3 Sample 1. The time evolution of the characteristic length scale $\lambda_a(t)$ is shown in Supplementary Fig. 8. For BCAT-3 (Supplementary Fig. 8(a)), it appears that higher colloid volume fractions ϕ_0 and polymer concentrations ρ are correlated with faster growth of the phase domains. Note that the colloid radius a and polymer radius of gyration δ are the same for all of these samples. In addition, the values of ϕ_0 and ρ are varied together between samples: specifically, both values are largest for Sample 1, followed by Sample 2, then Sample 6 and then Sample 4. Thus, it is unclear which of the two parameters has a greater influence on the coarsening rate, which appears to increase with both ϕ_0 and ρ in Supplementary Fig. 8(a).

For the BCAT-4 experiment (Supplementary Figure 8(b)), ϕ_0 and ρ are similarly decreased together going from Samples 1 through 3, and a and δ are the same for all samples (Table I in the Main Text). Samples 1 and 2 evidently follow the same trend as the BCAT-3 samples: specifically, the coarsening rate of Sample 1 is evidently higher than that of Sample 2, and ϕ_0 and ρ are larger for the former. On the other hand, Sample 3 does not follow this rule, as its coarsening rate is the largest but its ϕ_0 and ρ values are the smallest. It is unclear why this is the case, making this single sample an outlier.

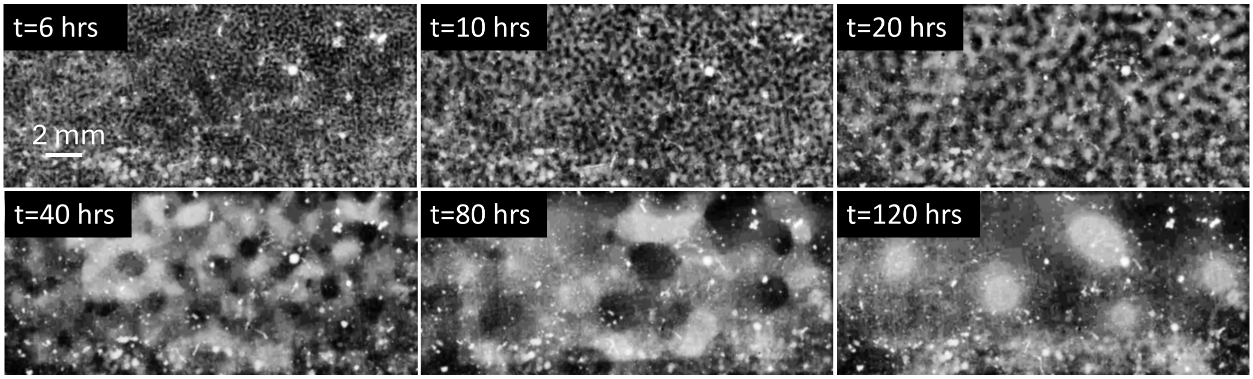

Supplementary Figure 1: Time evolution of BCAT-3, Sample 1. The parameters are listed in Table I in the Main Text.

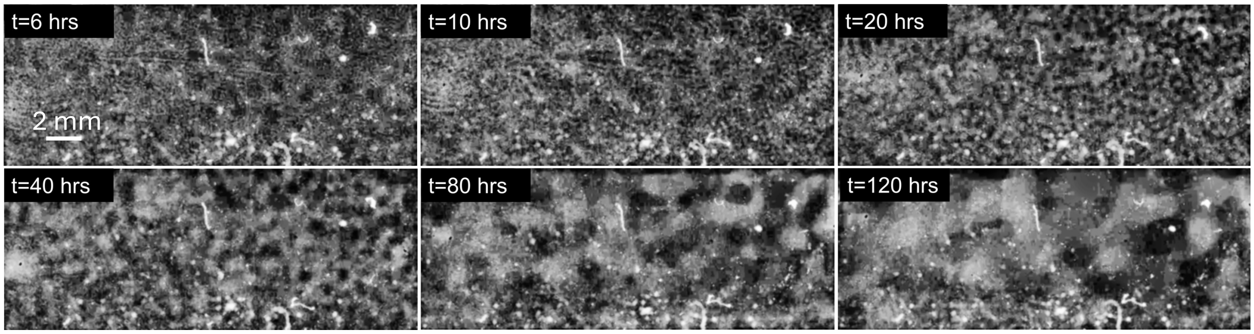

Supplementary Figure 2: Time evolution of BCAT-3, Sample 2. The parameters are listed in Table I in the Main Text.

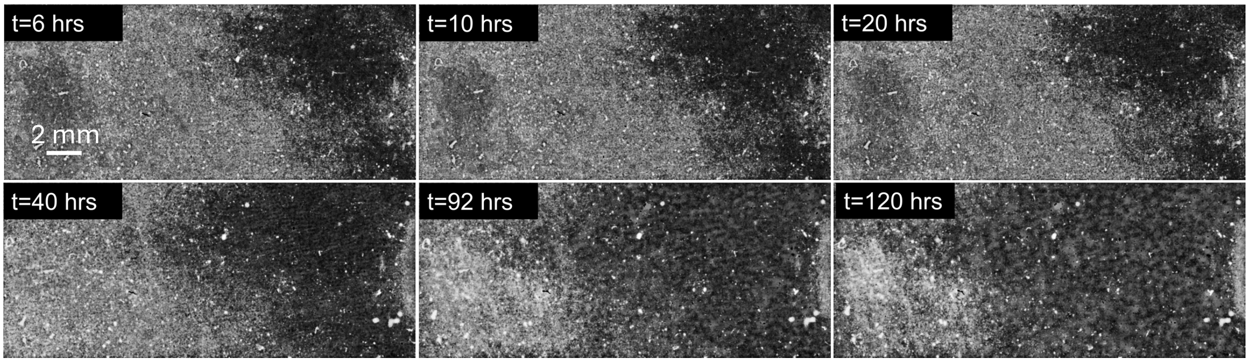

Supplementary Figure 3: Time evolution of BCAT-3, Sample 4. The parameters are listed in Table I in the Main Text.

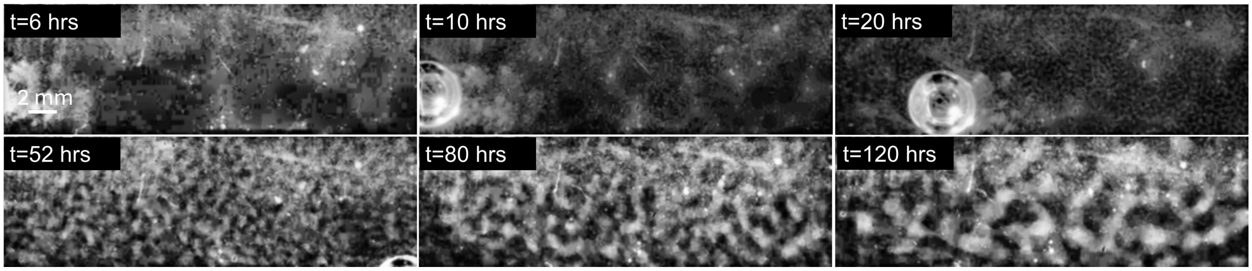

Supplementary Figure 4: Time evolution of BCAT-3, Sample 6. The parameters are listed in Table I in the Main Text. The circular object in the images taken at $t = 10$ hours and $t = 20$ hours is the magnetic stir bar used by the astronauts to mix the samples.

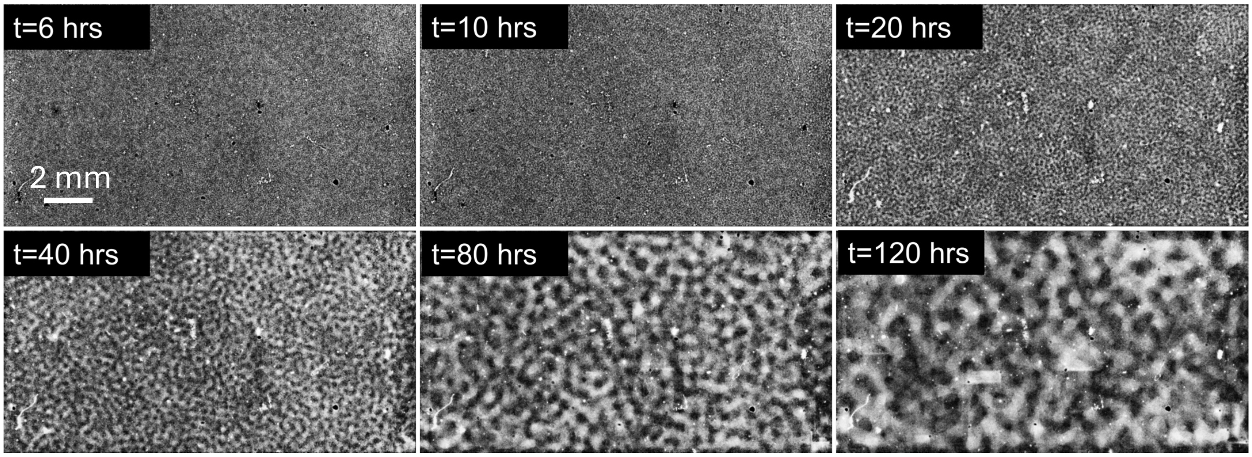

Supplementary Figure 5: Time evolution of BCAT-4, Sample 2. The parameters are listed in Table I in the Main Text.

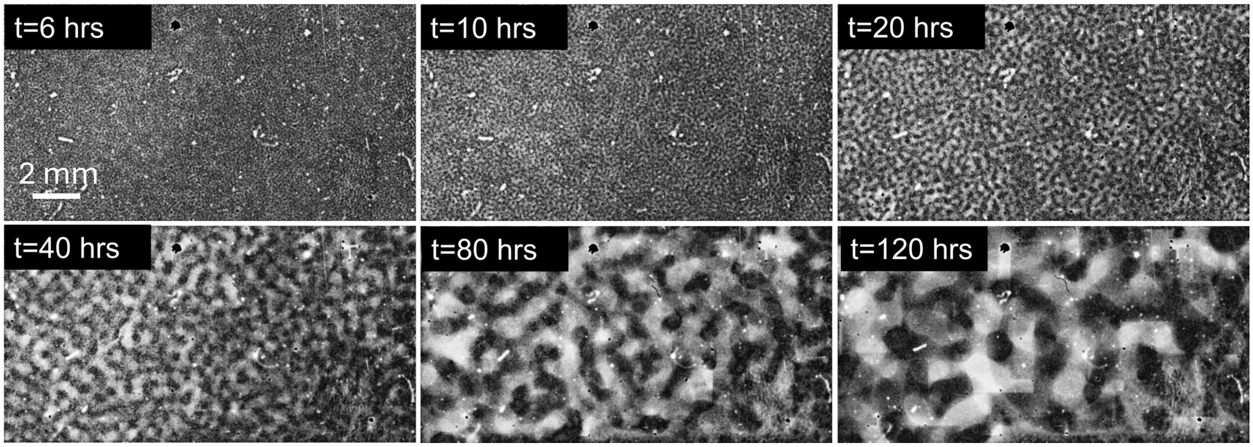

Supplementary Figure 6: Time evolution of BCAT-4, Sample 3. The parameters are listed in Table I.

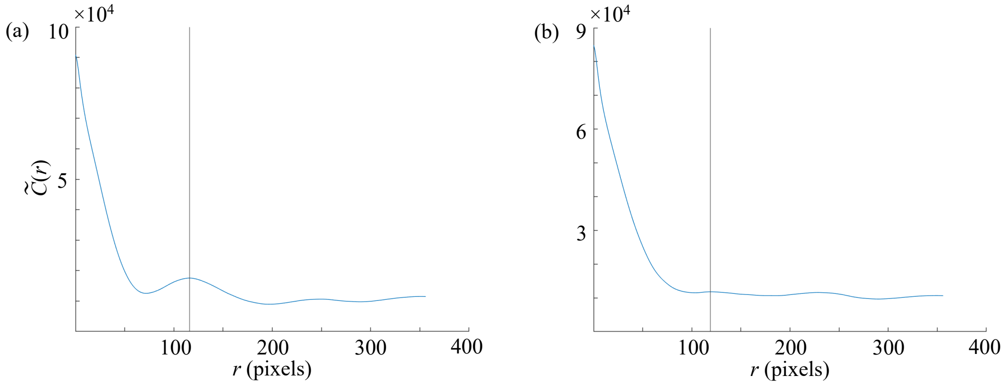

Supplementary Figure 7: Examples of the 1D autocorrelation function $\tilde{C}(r)$ for BCAT-3 Sample 1, at the times $t = 67$ hours (a) and $t = 86$ hours (b). The vertical gray lines indicate the location λ_a of the first maximum in $\tilde{C}(r)$ for $r > 0$. The value of λ_a is evidently slightly larger in (b) than (a), as one would expect for a coarsening process.

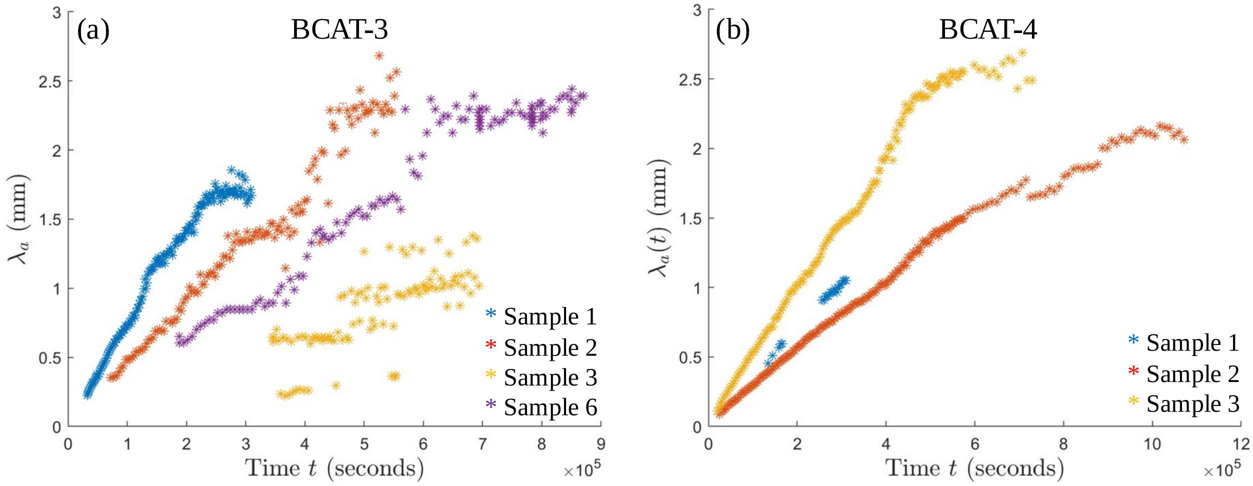

Supplementary Figure 8: Time evolution of the characteristic length scale $\lambda_a(t)$ for the samples in the BCAT-3 (panel (a)) and BCAT-4 (panel (b)) experiments. Note that comparatively few images were available for BCAT-4 Sample 1, likely because the camera malfunctioned during the experiment.

CAPTIONS FOR SUPPLEMENTARY MOVIES

Supplementary Movie 1: Simulation of BCAT-5 Sample 7, with $\gamma = 130$; the other parameters are listed in Table I in the Main Text. The left, middle and right panels correspond to the colloid volume fraction ϕ, fluid speed $|\gamma \boldsymbol{u}|$ and fluid vorticity $\gamma \boldsymbol{\nabla} \times \boldsymbol{u}$, respectively.

Supplementary Movie 2: Simulation of BCAT-5 Sample 4, with $\gamma = 93$; the other parameters are listed in Table I in the Main Text. The left, middle and right panels correspond to the colloid volume fraction ϕ, fluid speed $|\gamma \boldsymbol{u}|$ and fluid vorticity $\gamma \boldsymbol{\nabla} \times \boldsymbol{u}$, respectively.

Supplementary Movie 3: Simulations of BCAT-5 Sample 4 (top row) and BCAT-5 Sample 7 (bottom row), without (left column) and with (right column) hydrodynamics. The panels show the colloid volume fraction ϕ. While $\gamma = 0$ for the two videos in the first column, $\gamma = 93$ and $\gamma = 130$ for BCAT-5 Sample 4 and Sample 7, respectively.
